# WEIRD but Also Inconsistent: An Analysis of the Reporting Practices of Participant Samples Across Five Areas of Psychology

**DOI:** 10.1002/ijop.70168

**Published:** 2026-01-28

**Authors:** Leah Petrutiu, Megan E. Birney, Richard Cooke, Simon Stewart

**Affiliations:** ^1^ School of Health, Education, Policing, & Sciences, University of Staffordshire Stoke‐On‐Trent UK; ^2^ School of Education, University of Birmingham Birmingham UK

**Keywords:** generalisability, methodology, under‐representation, WEIRD science

## Abstract

In this study, we systematically investigate the Methods sections of five journals covering core areas of Psychology: Social, health, clinical, developmental, and general psychological science. Journals were published by the *British Psychology Society* between January 2021 and December 2023 (*N* ^articles^ = 661; *N* ^samples^ = 1293). As expected, we found an over‐reliance on Western perspectives: Participants from Latin America, Eastern Europe, the Middle East, and Africa made up 8.7% of samples combined. However, we also found substantial variation in whether and where participants' gender, race, SES indicators, and education were reported across different areas of Psychology, as well as different norms in the use of students and crowd‐sourcing platforms. Given the challenges of representation in Psychology and the importance of interdisciplinary perspectives, we make a case for a unified standard of reporting that allows readers to more readily access how findings generalise to populations beyond those sampled.

## 1 Introduction

Since Henrich et al.'s (2010) coining of the term WEIRD to describe the over‐reliance on Western and Educated participants from Industrialised, Rich, and Democratic countries used in behavioural research, assessments of representation in Psychology has mostly focused on journals published in the United States. For instance, an analysis of *Psychological Science* in 2017 found that over 70% of participant samples came from WEIRD populations (Rad et al. 2018). In top journals published by the American Psychological Association (APA) an over‐reliance on WEIRD samples between 2014 and 2018 meant that only 11% of the world's population was represented (Thalmayer et al. 2021). Indeed, studies from the U.S. consistently show that WEIRD participants make up between 70% and 90% of samples across psychology and the behavioural sciences (see Pollet et al. 2024 for a review).

Even within this narrow slice of humanity, many participants are recruited from universities in the U.S. (Apicella et al. 2020) and specifically, from introductory psychology courses (Sears 1986), further narrowing the scope of these samples away from the general population. In addition to being WEIRD, when compared to the general non‐student adult WEIRD population, these participants tend to be younger and more easily influenced (Sears 1986), rate higher on dimensions of individualism (Snibbe and Markus 2005), be more likely to mask negative attitudes towards outgroups (Henry 2008), and to have less tightly embedded social networks (Falk et al. 2010). In the context of the human population, this group is such an extreme outlier in the ways they think, feel, and behave that findings using these participants are often not generalisable to other Americans, much less to non‐WEIRD groups (Henrich et al. 2010).

Over the past 15 years, Psychology has taken steps to address underrepresentation. Henrich et al.'s (2010) coining of the term WEIRD and review of variation across human populations served as a wake‐up call, challenging the field's assumption that data collected from small samples of American undergraduates generalise to all human beings. At the time of writing, their paper has been cited over 17,390 times and inspired numerous studies to consider how humans differ across populations (Apicella et al. 2020). Researchers have also called for caution when generalising findings from WEIRD samples (Simons et al. 2017) and when applying them to policy (IJzerman et al. 2020). More value is being placed on qualitative methods for accessing under‐represented groups (Arellano 2022), and secondary data‐sources for understanding the influence of culture on behaviour (Muthukrishna et al. 2020). Various recommendations for increasing diversity in research have also been made (e.g., Amir and McAuliffe 2020; Henrich et al. 2010; Rad et al. 2018) from teaching the importance of culture in undergraduate psychology courses to encouraging international research collaborations (Thalmayer et al. 2021).

There has been a positive response from journals and psychology societies to these initiatives: APA publishing guidelines now include a focus on ‘equitable content’ (APA 2024) and, to increase representation among scholars, publishers have started gathering demographic data from their authors (see Else and Perkel 2022). Editors of two leading Psychology journals in the U.S., *Psychological Science* and the *Journal of Personality and Social Psychology*, and the president of the Association for Psychological Science have made statements explicitly encouraging ‘diverse’ submissions in terms of samples and authorship (Apicella et al. 2020). Task forces and professional organisations have also been established, and various special issues, guidelines, and strategic plans have been written to address under‐representation.

Despite these efforts, progress remains slow. For instance, although the use of student samples has declined in the years between 2005 and 2018, this is likely due to the emergence of online crowdsourcing platforms (e.g., MTurk, Prolific; Thalmayer et al. 2021), rather than sustained efforts to engage non‐student samples by diversifying recruitment methods. While crowdsourcing has been lauded as an effective way to reach underrepresented groups (Buhrmester et al. 2011; Pollet et al. 2024), it is unlikely that effects found in these samples can be generalised to larger groups of humans better than recruiting WEIRD samples; participants on crowdsourcing platforms tend to be white and, compared to the general populations they are drawn from (e.g., nationalities), younger, more educated, unmarried, more liberal, less religious and less employed (Schimmelpfennig et al. 2024). Furthermore, in their analysis of APA journals in the years succeeding Henrich et al.'s (2010) seminal paper, Thalmayer et al. (2021) reported an approximate 10% decrease in the use of American samples and publications by American authors over a 10‐year period. However, this change was due to increases from other English‐speaking and Western European countries, doing little to address Psychology's overall ‘WEIRDness’.

Given the dominance of American participants, and particularly American undergraduate students in psychological research, it is understandable that systematic reviews of participant and author representation have centred on journals published in the United States. However, to tackle issues resulting from unrepresentation, it is important to consider the norms of other countries that contribute to knowledge of human behaviour. In the current study, we investigate sample and author diversity, as well as breadth and consistency in reporting participant demographics in the United Kingdom (UK). Like the U.S., the UK is a WEIRD country. It is also a Psychology powerhouse with its own training programmes, services, and governing body, The British Psychological Society (BPS). Like the role of the APA in the U.S., the BPS represents UK psychology, taking ‘national responsibility for the development, promotion, and application of psychology for the public good’ (Science Council 2024). It has also made a commitment to tackling the dominance of WEIRD perspectives, both through its Equality, Diversity and Inclusion Framework, and its recent policy of requesting Diversity Data Statements from authors (Rhodes 2024).

In this study, we conduct a systematic investigation of five journals published by the BPS. Like the APA journals analysed by Thalmayer et al. (2021), these are highly cited and could be considered gatekeepers in their respective areas of Psychology (Arnett 2008). First, our aim is to understand the diversity of author and participant samples across these journals. Given the high status of the BPS within UK Psychology, we expect that the UK (rather than the U.S.) will be the most represented country in terms of both authorship and participant sample. However, because of its relationship with Western Europe and other English‐speaking countries, we expect that, like its American counterparts, Western perspectives will dominate.

Second, we aim to gain insight into norms of reporting key participant characteristics across different areas of Psychology. In line with Rad et al. (2018), we coded whether authors report their samples' gender identity, socio‐economic status (SES), race/ethnicity, and education level. We also coded whether samples were recruited via crowdsourcing and whether they consisted of university students. We gather this information to help us understand current norms around transparency in reporting participant demographics, and to gain insight into the use of university student and crowdsourcing across different areas of Psychology.

## 2 Method

This study was pre‐registered. Details of this, and the analysis, can be found on the OSF project page (https://osf.io/6bfze/?view_only=e791610afe5f461c86cf4b5b091b077b). See also the Supporting Information for the PRISMA chart for this study (Petrutiu et al. 2026).

Five flagship BPS journals were included in the analysis: *British Journal of Psychology, British Journal of Clinical Psychology, British Journal of Developmental Psychology, British Journal of Health Psychology, and British Journal of Social Psychology*. These journals were chosen because they cover core topics in Psychology and have influence in their respective fields (e.g., impact factors range from 2.6. to 3.8 and four of these are in the top quartile of QS impact journals, as of 2024). Across these journals, a total of 916 papers were identified between January 2021 and December 2023. Of these, six were duplicates and hence removed from the analysis. The study focused on quantitative studies because this methodology's focus on generalisability means it relies on samples that reflect the overall population (Parsons et al. 2024). Of the 910 papers screened for inclusion, 249 were removed for not using quantitative methods. In line with previous literature (e.g., Thalmayer et al. 2021; Rad et al. 2018), studies consisting of participant samples from more than one country were counted for each country represented. For example, if a study included participants from three different countries, it was recorded as three separate samples. The final dataset consisted of 661 papers, consisting of 722 studies that reported 1293 samples.

In line with Thalmayer et al. (2021), national affiliation was recorded for first and second authors, and for the participant sample in each paper. National affiliations of authors and participants were recorded by country (of each author's institution and the reported country that participants were recruited from) and these were then coded by region: West, Asia, Eastern Europe, Latin America, the Middle East, and Africa.^1^ In cases where no information was provided about participants' nationality or where they were recruited from, these were coded as ‘unspecified’ (see the OSF page for more detailed criteria for each measure and the individual articles that made up the sample). The use of university student samples was recorded for each study as 1 (only students), 2 (no students), or 3 (a mix of students and non‐students). The use of crowdsourcing in each study was recorded as yes or no. Finally, whether authors reported their sample's demographic characteristics for each study (i.e., gender, education level, socio‐economic status, and race) was recorded as yes or no (see Rad et al. 2018). During data collection, it became apparent that there was inconsistency as to where in the manuscript this information could be found. As APA formatting stipulates that participant sample information in quantitative papers should be found in a study's Methods section (American Psychology Association 2020), whether this information could be found in this particular section was also recorded (as yes or no; see the OSF project page).

## 3 Results

All data can be found in Tables 1, 2, 3, 4.

**TABLE 1:** The top 10 national affiliations of authors (by paper) and participants (by study).

	First authors		Second authors		Participants	
Country^a^	%	*n*	%	*n*	%	*n*
UK	25	165	24.7	159	20	258
USA	13.9	92	16.1	104	17.7	229
Australia	9.2	61	9.8	63	3.9	51
China	7.6	50	7.1	46	6.8	88
Germany	7.4	49	8.1	52	5.5	71
Canada	4.5	30	4.8	31	3.9	51
Italy	3.9	26	3.9	25	2.2	29
Netherlands	3.9	26	4.8	31	2.5	32
Switzerland	2.3	15	1.7	11	0.9	12
Spain	2.1	14	2.8	18	3.2	41
France	2.1	14	1.9	12	2.1	27
Poland	2.1	14	1.1	7	1.9	25

^a^ List is in the chronological order of the first author data.

**TABLE 2:** First and second author's regional affiliation.

	Author 1 (*n* = 661)						Author 2 (*n* = 644)					
	West	Asia	Latin America	Eastern Europe	Middle East	Africa	West	Asia	Latin America	Eastern Europe	Middle East	Africa
	% (*n*)	% (*n*)	% (*n*)	% (*n*)	% (*n*)	% (*n*)	% (*n*)	% (*n*)	% (*n*)	% (*n*)	% (*n*)	% (*n*)
Sample Overall	82 (542)	10.7 (71)	1.1 (7)	4.2 (28)	1.8 (12)	0.2 (1)	84.5 (544)	9.6 (62)	0.8 (5)	2.8 (18)	2.0 (13)	0.3 (2)
*British Journal of Psychology* (A^1^ *n* = 148; A^2^ *n* = 142)	73.6 (109)	20.3 (30)	0 (0)	4.1 (6)	2.0 (3)	0 (0)	78.2 (111)	16.9 (24)	0 (0)	2.8 (4)	2.1 (3)	0 (0)
*British Journal of Clinical Psychology* (A^1^ *n* = 120; A^2^ *n* = 120)	91.7 (110)	0.8 (1)	1.7 (2)	3.3 (5)	2.5 (3)	0 (0)	94.2 (113)	0.8 (1)	0.8 (1)	1.7 (2)	2.5 (3)	0 (0)
*British Journal of Developmental Psychology* (A^1^ *n* = 94; A^2^ *n* = 92)	79.8 (75)	12.8 (12)	2.1 (2)	4.3 (4)	1.1 (1)	0 (0)	80.4 (74)	12 (11)	2.2 (2)	3.3 (3)	2.2 (2)	0 (0)
*British Journal of Health Psychology* (A^1^ *n* = 116; A^2^ *n* = 113)	87.9 (102)	6.9 (8)	0 (0)	3.4 (4)	0.9 (1)	0.9 (1)	91.2 (103)	4.4 (5)	0 (0)	2.7 (3)	0.9 (1)	0.9 (1)
*British Journal of Social Psychology* (A^1^ *n* = 183; A^2^ *n* = 177)	79.8 (146)	10.9 (20)	1.6 (3)	5.5 (10)	2.2 (4)	0 (0)	80.8 (143)	11.9 (21)	1.1 (2)	3.4 (6)	2.3 (6)	0.6 (1)

*Note:* The sample size for manuscripts with (at least) one author is indicated by A^1^. Sample sizes for manuscripts with (at least) two authors are indicated by A^2^.

**TABLE 3:** Participants' regional affiliation.

	Participant sample by region							
	West	Asia	Latin America	Eastern Europe	Middle East	Africa	Not specified	Large scale dataset
	% (*n*)	% (*n*)	% (*n*)	% (*n*)	% (*n*)	% (*n*)	% (*n*)	% (*n*)
Sample Overall (*n* = 1293)	67.6 (874)	9.9 (128)	1.7 (22)	4.4 (57)	1.4 (18)	1.2 (15)	13.1 (170)	0.7 (9)
*British Journal of Psychology* (*n* = 329)	58.4 (192)	10.3 (34)	0 (0)	2.1 (7)	1.2 (4)	1.5 (5)	25.5 (84)	0.9 (3)
*British Journal of Clinical Psychology* (*n* = 150)	80 (120)	2.7 (4)	2.7 (4)	3.3 (5)	2.7 (4)	0 (0)	8.7 (13)	0 0
*British Journal of Developmental Psychology* (*n* = 116)	62.9 (73)	13.8 (16)	8.6 (10)	3.4 (4)	1.7 (2)	1.7 (2)	7.8 (9)	0 0
*British Journal of Health Psychology* (*n* = 181)	81.2 (147)	7.7 (14)	0 0	1.7 (3)	0 0	2.2 (4)	7.2 (13)	0 0
*British Journal of Social Psychology* (*n* = 517)	66.2 (342)	11.6 (60)	1.5 (8)	7.4 (8)	1.5 (8)	0.8 (4)	9.9 (51)	1.2 (6)

**TABLE 4:** Participant demographics.

	Student sample			Crowdsourced	Participant demographic reported (yes)				Reported in methods
	Yes	No	Mix	Yes	Gender	Education level	SES	Race	Yes
	% (*n*)	% (*n*)	% (*n*)	% (*n*)	% (*n*)	% (*n*)	% (*n*)	% (*n*)	% (*n*)
Sample overall (*n* = 1293)	18.9 (245)	73.5 (950)	7.6 (98)	36.6 (473)	94.8 (1126)	53 (685)	26.5 (343)	36.7 (475)	86.9 (1122)
*British Journal of Psychology* (*n* = 329)	34.3 (113)	59.6 (196)	6.1 (20)	31.9 (105)	88.8 (292)	49.8 (164)	8.2 (27)	33.4 (110)	90.6 (298)
*British Journal of Clinical Psychology* (*n* = 150)	8.7 (13)	79.3 (119)	12 (18)	18 (27)	95.4 (143)	62.7 (94)	34 (51)	51.3 (77)	60 (90)
*British Journal of Developmental Psychology* (*n* = 116)	2.6 (3)	90.5 (105)	6.9 (8)	6 (7)	97.4 (113)	80.2 (93)	97.4 (113)	43.1 (50)	95.7 (111)
*British Journal of Health Psychology* (*n* = 181)	8.8 (16)	79.6 (144)	11.6 (21)	16 (29)	93.9 (170)	50.3 (91)	47 (85)	32 (58)	69.1 (125)
*British Journal of Social Psychology* (*n* = 517)	19.3 (100)	74.7 (386)	6 (31)	59 (305)	98.3 (508)	47 (243)	25.3 (131)	34.8 (180)	96.3 (498)

### 3.1 Authorship

The institutional affiliations of both first and second authors represented 42 countries. Approximately a quarter of each (25% of first authors and 24.7% of second authors) came from UK universities. However, there was a clear domination of researchers based on Western universities. The top 12 countries represented by authors (identified using their institutional affiliation) can be seen in Table 1: Of these, 10 were Western. China was the only country that disrupted the domination of Western perspectives (with 7.6% of first authors and 7.1% of second authors respectively). The other non‐Western country (Poland) was represented similarly frequently to France and Spain (2.1% respectively).

The dominance of authorship from the West is also reflected in the analysis of author affiliation by region: Here, 82% of first authors and 84.5% of second authors were affiliated with Western institutions. When looking at the regional affiliation of authors by journal, the *British Journal of Clinical Psychology* had the highest number of Western affiliated authors (91.7% of first authors and 94.2% of second authors) while the *British Journal of Psychology* had the lowest (73.6% and 78.2% of authors respectively; see Table 2).

### 3.2 Sample Characteristics

Across the 1293 samples analysed, most participants (67.6%) were recruited from Western countries (see Table 3). In Western samples, the UK was the most represented group (20%) with the U.S. following in second (17.7%). In total, the rest of the world made up 18.6% of participants across the sample, with almost a third of these (9.9%) coming from Asia. In 13.1% of studies, the country participants were recruited from was not specified. At first glance, it seems the *British Journal of Psychology* was the least Western with 58.4% of participants coming from this region (the *British Journal of Health Psychology* had the highest number of Western participants with 81.2%). However, this journal also had the highest number of instances where the country participants were recruited from was not reported (25.5%). The journal with the next highest percentage of participants where country of recruitment was unspecified was *British Journal of Social Psychology* at 9.9%.

Overall, 18.9% of studies across journals recruited only undergraduates (see Table 4). Studies in the *British Journal of Psychology* reported the highest percentage of student‐only samples (34.3%) while the *British Journal of Developmental Psychology* reported the lowest (2.6%). Data from th*e British Journal of Social Psychology* showed a fairly low number of student samples (e.g., 74.7% of studies contained no undergraduates at all), but this seemed to be due to the popular use of crowdsourcing in this journal, which was the highest percentage of all of the journals we analysed (59% of studies; see Table 2).

### 3.3 Reporting of Participant Demographics

Results demonstrated disparities across different areas of psychology in terms of where, and whether, key demographic information of participant samples was reported. Despite APA guidelines specifying that participant characteristics be reported in the Methods section (APA 2024), 13.1% of studies' Methods did not include this (see Table 4). This number was driven by the *British Journal of Clinical Psychology* and the *British Journal of Health Psychology* whose Methods sections only included participant demographics 60% and 69.1% of the time, respectively. The other three journals—*British Journal of Psychology, British Journal of Developmental Psychology, and British Journal of Social Psychology*—had rates of over 90%.

Demographic information was reported differently across journals (see Table 4). For instance, SES was reported 97.4% of the time in the *British Journal of Developmental Psychology* but only 8.2% in the *British Journal of Psychology* while the reporting of education level varied between 80.2% (*British Journal of Developmental Psychology*) and 47% (*British Journal of Social Psychology*). Participants' race was reported more consistently across journals but at lower rates: the *British Journal of Clinical Psychology* had the highest rate (51.3%) while the *British Journal of Health Psychology* had the lowest (32%). Gender had the highest reporting rates (94.8% overall) as well as the most consistent, ranging from 88.8% (*British Journal of Psychology*) to 98.3% (*British Journal of Social Psychology*).

## 4 Discussion

Like other behavioural sciences, Psychology continues to grapple with the over‐representation of WEIRD samples in their studies. However, most systematic investigations of Psychology journals have focused on research published in the United States. This study adds to this literature by investigating representation within Psychology in the UK to understand its contribution to the status quo. Through a systematic investigation of five key journals published by the British Psychological Society (BPS) over the 3 years from 2021 to 2023, we had two aims: (a) to investigate the diversity of perspectives represented across these journals and (b) to understand how key participant characteristics are reported across different areas of Psychology.

### 4.1 Author and Sample Characteristics

Our analysis showed that BPS journals are less ‘British’ than APA journals are ‘American’. In Thalmayer et al.'s (2021) analysis of key APA journals, 60% of authors and participants were American, while our analysis of key BPS journals found less than a quarter of these were from countries in the UK. However, this is not a direct comparison; the first year of Thalmayer et al.'s (2021) analysis took place 7 years before the first year we analysed (2014 vs. 2021). Given Psychology's efforts to reach underrepresented groups over this time (see Apicella et al. 2020), studies in APA journals published between 2021 and 2023 may be more diverse than they were between 2014 and 2018. Since Thalmayer et al. (2021) study found only a 10% decrease in ‘Americanness’ in the decade preceding their work (see Arnett 2008), it seems unlikely that these perspectives would be close to the level of UK perspectives we found (25% of first authors, 24.7% of second authors, and 20% of participants).

However, having fewer UK perspectives did little to quell the dominance of Western perspectives across our sample. Overall, 67.6% of participants were recruited from Western countries, 18.6% from non‐Western countries, while 13.1% of samples did not report this information. Given the tendency to leave groups considered normative unexplained (see Bruckmüller 2013), it is possible that many of these were also recruited in the West. If this is the case, then Western participants could make up as much as 80% of the participants in our sample. In terms of authorship, scholars from Western universities made up over 80% of first and second authors, and across all journals, at least 73% were Western. Scholars in Asia were the second most represented group (making up approximately 10% of first and second authors) meaning that the rest of the world (i.e., scholars from Latin America, Eastern Europe, the Middle East, and Africa) consisted of only 7.3% and 5.9% of first and second authors respectively. Like previous work (e.g., Arnett 2008; Thalmayer et al. 2021) author's nationality was inferred from their affiliated University. While this may not reflect authors' actual nationality, the fact that such a large percentage of publications come from Western institutions—which hold more research and cultural capital than non‐Western institutions—is further evidence of Psychology's WEIRDness. Scholars from around the world are, understandably, attracted and the prestige of these institutions (Abramo et al. 2019); with one analysis from 2004 finding that over 30% of academics are not working in the place of their birth (Ioannidis 2004). How scholars from non‐Western countries adapt to these environments has been explored (see Martin and Dandekar 2022). However, the effect this experience has on the knowledge they generate, and how their perspectives shape Western research, is an important area for further research.

As expected, we found a relatively small proportion of studies relied solely on undergraduate samples (18.9% across the five journals). This may reflect the foci of three of the journals we chose to review; the *British Journal of Developmental Psychology* mainly reports results from school‐age children, while the *British Journal of Clinical Psychology* and the *British Journal of Health Psychology* tend to report results from patient populations. However, the picture from Social Psychology, which has a history of relying on undergraduates as participants (Sears 1986), gives some insight into current practice. In line with Thalmayer et al. (2021), we found that the use of students appears to be declining (19.3% of studies in the *British Journal of Social Psychology* recruited student samples). While we do not have direct comparison data, an analysis of the same journal between 1990 and 2005, but that focused specifically on articles related to prejudice, found that 76.9% of studies relied solely on students (Henry 2008).

There are two likely reasons for the seemingly dramatic decrease in the use of student samples over the past three decades. First, since Henrich et al.'s (2010) seminal paper highlighting the overuse of WEIRD perspectives in the behavioural sciences, journals have placed greater value on studies that recruit from outside of this group (including discouraging the use of students; Apicella et al. 2020). For instance, the *British Journal of Health Psychology* adopted an editorial position that they did not want to publish studies that rely solely on student samples (BPS 2025). Also, all journals published by the BPS include an EDI Statement of Commitment. While there is not an explicit statement on the use of students, there is a commitment to ‘Ensure our journals offer a diverse range of content and voices’ (Rhodes 2024). These positions, as well as the greater acknowledgment of issues associated with the dependency on students (Apicella et al. 2020), mean researchers are increasingly being challenged to find ways to increase representation within their samples.

The pressure to diversify samples has also coincided with the development of sophisticated online data collection tools (e.g., Qualtrics) and online crowdsourcing platforms (e.g., MTurk, Prolific; Buhrmester et al. 2018). As such, a second reason for the decline in the use of students is that researchers, especially Social Psychologists, appear to have turned to online data collection for convenience sampling (see also Thalmayer et al. 2021). Our analysis showed that crowdsourced participants made up 59% of samples in the *British Journal of Social Psychology*, and 36.6% of the sample overall. While there are many benefits to recruiting participants this way, including increased access to under‐represented groups (Pollet et al. 2024), concerns have been raised about how representative users are of the places they are recruited from (Schimmelpfennig et al. 2024). Research on the users of these platforms, both in terms of their characteristics and their motivations, is still in its infancy. Given the growing popularity of this recruitment method, more research is needed into (a) how well they represent their overall group (in terms of nationality, gender, etc.) and (b) how to leverage this technology to increase representative and participant diversity.

### 4.2 Reporting of Participant Demographics

Our analysis showed a lack of consistency in how samples were described. Gender identity was the demographic most consistently included in authors' descriptions of their participants, with 94.8% of studies reporting this. Yet, whether education level was reported was just over chance (53% of studies overall), while race and SES were reported only 36.7% and 26.5% of the time respectively. The norm of reporting gender may reflect the increasing inclusion of women in psychological research over the past 60 years, while researchers (and journals) do not seem to have caught up with the inclusion of other demographic groups (e.g., participants of varying education levels, ethnicities, and economic backgrounds). However, these demographics are key to understanding how results should be interpreted and whether they are likely to generalise to wider populations. For instance, participants from less WEIRD countries that have higher levels of formal education have been found to be more culturally similar to participants in the U.S. (White and Muthukrishna 2023). If we want to interpret our data in ways that are meaningful, we need to understand the context in which that data was collected. This starts with recording and reporting key characteristics of participants in our studies.

In addition to inconsistencies in what was reported, there was variation across journals regarding where participant information was reported. Despite APA guidance stipulating these be in the Methods section of a manuscript (American Psychological Society 2020), we found that whether this happened ranged between 60% of the time (in the *British Journal of Clinical Psychology*) to 96.3% of the time (in the *British Journal of Social Psychology*). Of course, it is possible that this information was reported in other sections of the manuscript (i.e., the results or appendices). While different norms across a field as diverse as psychology are inevitable, where and what participant information can be found should be consistent. Ensuring that APA formatting is more strictly adhered to, in terms of location in the manuscript and the demographics reported, would encourage psychologists to comment on the representativeness of their samples with more transparency. It would also allow readers to draw conclusions and to make connections more readily. Information that is missing or difficult to find adds what seems an unnecessary hurdle to our field's goal of producing worthwhile and inclusive science.

Finally, while our analysis focused on whether key demographic information was reported, future work should focus on the representation of participants across these demographics. For instance, of the just over a quarter of samples that reported SES, knowing the range of SES represented would add further insight into where we are, as a field, in reaching diverse groups of humans. It would also help us to acknowledge the role that power structures play in terms of who we base knowledge on, and who is left out. An analysis at this level would add much needed nuance to the conversation on representation, beyond the countries participants are from.

## 5 Conclusion

In a 1946 opinion piece published in *Psychological Bulletin*, the author stated that ‘the existing science of human behaviour is largely the science of the behaviour of sophomores’ (McNemar 1946, 333). While this is less true today, the fact that the majority of perspectives and samples are still WEIRD shows that our samples have not widened as much as we might have hoped in almost 80 years. What has changed is that researchers and journal editors are more aware of the implications of this, both in terms of the limitations it places on knowledge and the consequences it has for under‐represented groups. While this is an important development, it has not equated to increased representation to the same degree. Undoubtedly, there are many barriers to reaching under‐represented groups, regardless of how aware one is of its importance. For scholars in Western institutions who lack funding, recruiting from hard‐to‐reach populations can be difficult. Even when such populations are reached, say through crowdsourcing, there are issues with self‐selection bias. As a field, Psychology has some way to go to understand how to make our science truly representative of human beings.

In the meantime, one step we can take that is within our control is to ensure we are upfront and transparent about the samples we use and what it means for the knowledge we generate. We are certainly not the first researchers to make this point (see Rad et al. 2018 for a similar argument), and as a field, we are not alone in dealing with inconsistent reporting (see Parsons et al. 2024 that address similar issues in Radiology). Regardless, our data suggest that the point bears repeating. We call on researchers to record and to report key demographic information for all of their participants. At a minimum, this should include age, gender, nationality, race/ethnicity, socio‐economic status, and education. Journals should also require this information and ensure that it is easy for readers to find and to navigate. By giving the information needed to make conjectures about whether and how our results generalise to other populations, we leave space for the voices who, for now at least, remain unspoken.

## Author Contributions

**Leah Petrutiu:** conceptualization, methodology, investigation, project administration, data curation, formal analysis, visualization, writing – review and editing. **Megan E. Birney:** conceptualization, methodology, writing – original draft, supervision. **Richard Cooke:** writing – review and editing, supervision. **Simon Stewart:** writing – review and editing, supervision.

## Funding

The authors have nothing to report.

## Ethics Statement

The authors have nothing to report.

## Conflicts of Interest

The authors declare no conflicts of interest.

## Supporting information


**Data S1:** The PRISMA chart.

## Data Availability

The data that support the findings of this study are openly available in Open Science Framework at https://osf.io/6bfze/?view_only=e791610afe5f461c86cf4b5b091b077b.

## Endnotes

^1^
Regions were coded as follows: West = UK, USA, Canada, Italy, Greece, Germany, Denmark, Australia, Spain, Netherlands, Ireland, France, Switzerland, Belgium, Sweden, Norway, New Zealand, Finland, Austria, Portugal. Asia = China, Singapore, South Korea, Malaysa, Japan, Hong Kong, Thailand, Kyrgyzstan, India, Brunei, Pakistan, Philippines, Indonesia, UAE; Latin America = Latin America, Brazil, Argentina, Chile, Mexico, Uruguay, Venezuela, Bolivia, Peru, Paraguay, Ecuador, Barbados; Eastern Europe = Hungary, Turkey, Poland, Serbia, Croatia, Estonia, Kosovo, Russia, North Macedonia, Bosnia, Bulgaria, Latvia, Lithuania; Middle East = Iran, Israel, Lebanon, Egypt; Africa = South Africa, Nigeria, Ghana, Morocco, Namibia.
