# Supplementary material for: WEIRD but Also Inconsistent: An Analysis of the Reporting Practices of Participant Samples Across Five Areas of Psychology
Source: Int J Psychol. 2026 Jan 28;61(2):e70168. doi: 10.1002/ijop.70168 (PMC12852971; doi:10.1002/ijop.70168)
Supplement: Supplementary file 1 — Data S1: The PRISMA chart. [file IJOP-61-e70168-s001.docx]

**Identification**

Records identified

(Jan 2021 - Dec 2023) (n = 916)

Duplicates removed:(n = 6)

Marked ineligible by automation: (n = 0)

Removed for other reasons: (n = 0)

BPS Journals selected:

British Journal of Psychology

British Journal of Clinical Psychology

British Journal of Developmental Psychology

British Journal of Health Psychology

British Journal of Social Psychology

(n = 5)

**Screening**

Records screened at title:

(n = 910)

Records excluded

(n = 249)

Records excluded

(n = 0)

Records screened at abstract

(n = 661)

**Eligibility**

Full text articles excluded according to identification criteria

(n = 0)

Full text articles assessed for eligibility

(n = 661)

**Included**

Papers included in review

(n = 661)

Studies included in review

(n = 722)

Samples reported in review

(n = 1,293)

**PRISMA chart of study inclusion process.** Due to the nature of this research, all papers that passed abstract screening met the inclusion criteria for this study. Records were identified from five British Psychological Society journals published between January 2021 and December 2023, covering core areas of psychology. A total of 916 records were identified, of which 6 duplicates were removed. Subsequently, 910 records were screened at the title stage, with 240 records excluded. At the abstract screening stage, 661 records were assessed, with no exclusions. Full-text articles were then assessed for eligibility, totalling 661, with no reports excluded post-screening. The final dataset included 722 studies within these 661 papers, reporting on 1,293 participant samples. This approach aligns with prior literature (Thalmayer et al., 2021; Rad et al., 2018) *Note.* See OSF for DOI of each empirical paper included in the review.
